# The life of the freshwater bryozoan *Stephanella hina* (Bryozoa, Phylactolaemata)—a crucial key to elucidating bryozoan evolution

**DOI:** 10.1186/s40851-016-0060-5

**Published:** 2016-12-08

**Authors:** Thomas Schwaha, Masato Hirose, Andreas Wanninger

**Affiliations:** 1 Department of Integrative Zoology, University of Vienna, Althanstraße 14, Vienna, 1090 Austria; 2 Atmosphere and Ocean Research Institute, University of Tokyo, 5-1-5 Kashiwanoha, Kashiwa-shi, Chiba 277-8564 Japan

**Keywords:** Ectoprocta, Stephanellidae, Phylactolaemata, Ectocyst evolution, Solitary zooids

## Abstract

**Background:**

Phylactolaemata is the earliest branch and the sister group to all extant bryozoans. It is considered a small relict group that, perhaps due to the invasion of freshwater, has retained ancestral features. Reconstruction of the ground pattern of Phylactolaemata is thus essential for reconstructing the ground pattern of all Bryozoa, and for inferring phylogenetic relationships to possible sister taxa. It is well known that *Stephanella hina*, the sole member of the family Stephanelllidae, is probably one of the earliest offshoots among the Phylactolaemata and shows some morphological peculiarities. However, key aspects of its biology are largely unknown. The aim of the present study was to analyze live specimens of this species, in order to both document its behavior and describe its colony morphology.

**Results:**

The colony morphology of *Stephanella hina* consists of zooidal arrangements with lateral budding sites reminiscent of other bryozoan taxa, i.e., Steno- and Gymnolaemata. Zooids protrude vertically from the substrate and are covered in a non-rigid jelly-like ectocyst. The latter is a transparent, sticky hull that for the most part shows no distinct connection to the endocyst. Interestingly, individual zooids can be readily separated from the rest of the colony. The loose tube-like ectocyst can be removed from the animals that produces individuals that are unable to retract their lophophore, but merely shorten their trunk by contraction of the retractor muscles.

**Conclusions:**

These observations indicate that *S. hina* is unique among Phylactolaemata and support the notion that bryozoans evolved from worm-like ancestors. In addition, we raise several arguments for its placement into a separate family, Stephanellidae, rather than among the Plumatellidae, as previously suggested.

**Electronic supplementary material:**

The online version of this article (doi:10.1186/s40851-016-0060-5) contains supplementary material, which is available to authorized users.

## Background

Bryozoa or Ectoprocta is a group of sessile, colonial filter-feeders of approximately 6000 extant species. Its phylogenetic relationship to other lophotrochozoan taxa remains controversial (e.g. [1–4]). Due to shared morphological features in their general organization, including their feeding apparatus, the lophophore, this group was traditionally united with the Brachiopoda and Phoronida as ‘Lophophorata’ or ‘Tentaculata’ [5]. However, many molecular phylogenies have failed to support the ‘Lophophorata’ concept. Most have supported instead a close relationship between phoronids and brachiopods (e.g. [3, 6]), whereas only few recent studies have lent support to the ‘lophophorate’ grouping [7–9].

Bryozoans typically consist of several individuals, called zooids, which constitute the colony. Each zooid is typically divided into the cystid, which constitutes the body-wall, which is fortified with different extracellular secretions, and the polypide, which consists of the soft-body parts. Bryozoa can be divided into three class-level taxa: Phylactolaemata, Stenolaemata and Gymnolaemata. Morphological and molecular analyses agree that Phylactolaemata is the basal-most branch and sister-group to the remaining taxa [10–12]. Phylactolaemata is a small group of approximately 80 species worldwide within six families that only occur in freshwater habitats [13]. Their members show different variations of the ectocyst, which may be either chitinous or gelatinous. Various analyses agree that Stephanellidae with the sole currently recognized species *Stephanella hina* Oka, 1908 is one of the earliest branches among the Phylactolaemata [14]. This species is almost exclusively reported from Japan and Southeast Asia [15, 16] and the east coast of North America [17], and it shows a unique colony morphology with stolon-like connections and lateral budding loci [18]. In addition, its statoblasts and in particular the formation of the sessoblasts seems to differ from all other phylactolaemate bryozoans [19]. This species is also unique in that it starts to grow in December and starts to decay in mid-April when other phylactolaemates start to thrive after overwintering [20]. Only few other species are known to survive cold winter temperatures as adults [13].

Surprisingly, almost no data are currently available for this species; even photographic documentation is almost entirely lacking [16, 18, 21]. In addition, whether *Stephanella* constitutes a separate family-level taxon or is part of the Plumatellidae, the largest of all phylactolaemate families, remains unclear. Our aim in the present study was to identify and document the biology of this neglected species. As the earliest branch, the reconstruction of the phylactolaemate body plan may yield essential information into the evolution of the whole phylum, as well as the evolution of coloniality among metazoans. Bryozoa is the only animal phylum in which the members are all colonial. However, since all other possible lophotrochozoan outgroups have a solitary ground pattern, it is clear that the pre-bryozoan ancestor was also a solitary, probably worm-shaped, organism. Hence the elucidation of the phylactolaemate morphological ground pattern could yield insight into the evolution of solitary into colonial forms.

## Methods

Samples were collected in April 2014 and February 2015 and 2016 in Japan, either in the pond of the campus of the University of Tsukuba or in a freshwater pond in Tsuchiura-shi in close vicinity to the campus. Samples were collected from mainly wooden substrates and transferred into the laboratory for further inspection and treatment. Plants and artificial substrates (mainly garbage, shoes, etc.) were not colonized by any specimens. Living specimens were documented and filmed with a Nikon J1 camera (Nikon, Tokyo, Japan) mounted on a c-mount on a Nikon SMZ 1500.

## Results & discussion

### General structure of the colony and zooids

**Fig. 1:**
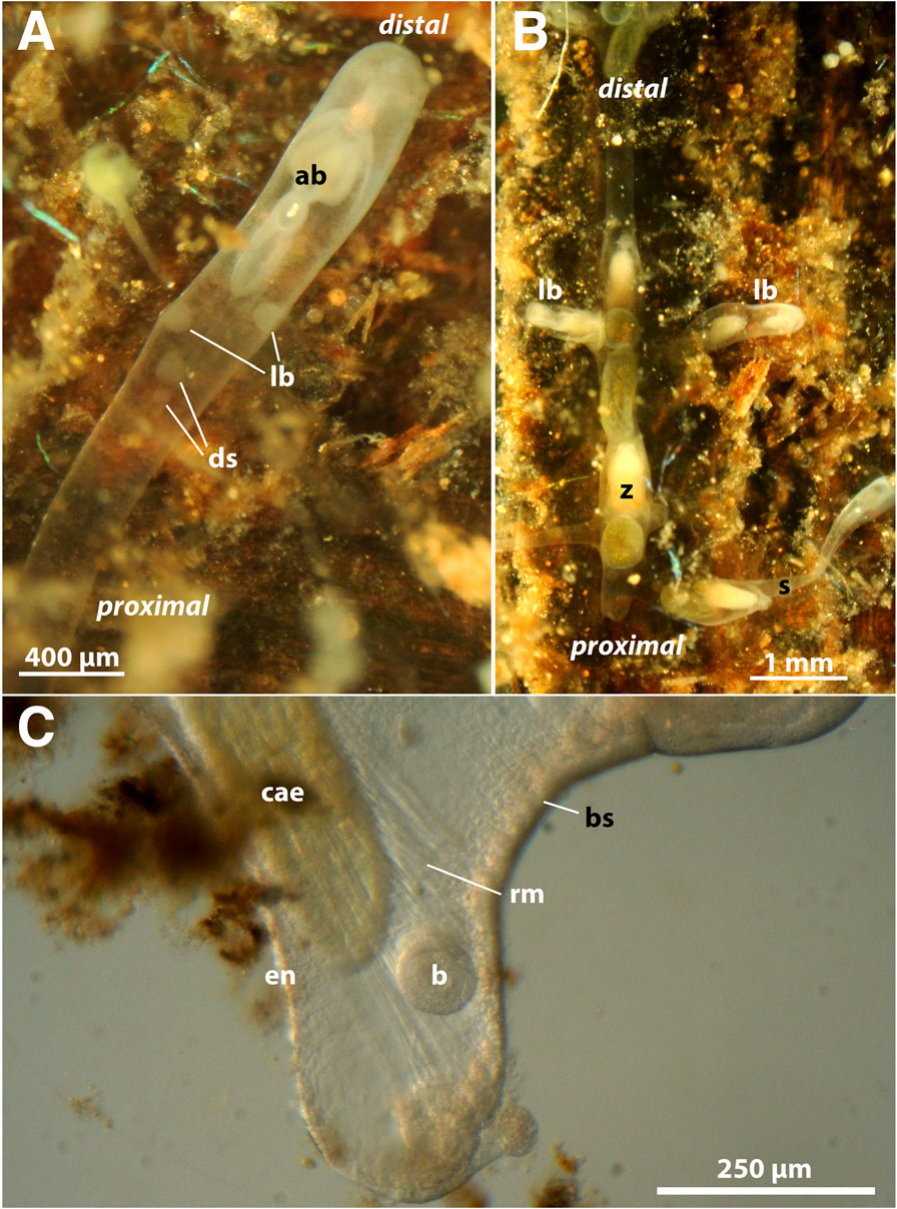
Budding sites of *Stephanella hina*. **a** View of an elongated distal bud, with two lateral buds and developing statoblasts. **b** Parts of a retracted colony, showing the cruciform growth of the colonies due to lateral budding. **c** Close-up of the basal part of a detached, single zooid, showing an early two-layered bud at its base close to the retractor muscle. Abbreviations: ab – advanced bud, b – bud, bs – basal attachment side, cae – caecum, ds – developing statoblasts, en – endocyst, lb – lateral buds, rm – retractor muscle, s – stolon, z – zooid

*Stephanella hina* colonies form an array of interconnected zooids, with each zooid generally having up to four connections to other zooids within the colony (Fig. 1a and b). These interconnections are restricted to the substrate and are, particularly in older colonies, stolon-like. Younger colonies commonly show a wider interzooidal connection. This corresponds to previous descriptions [18, 21] and is unique for a phylactolaemate bryozoan where zooids are normally interconnected by much wider spaces than in *Stephanella*. In this respect a similar tube-like connection is only found in the plumatellid genus *Stolella* [22, 23]. However, in the latter these are not as pronounced as in *S. hina*. Since there are three budding loci—one distal and two lateral—a total of four stolon-like connections are present in *S. hina* (Fig. 1a, b). Lateral buds are not present in any other phylactolaemate. This simple runner-like colony shape is also characteristic of early fossil representatives as well as recent, probably early branching, taxa of the other two bryozoan taxa Stenolaemata (e.g. Corynotrypidae) [24–26] and Gymnolaemata (e.g. *Arachnidium* and *Cardoarachnidium* in Ctenostomata [27]; e.g. *Pyripora* and *Pyriporopsis* in Cheilostomata [28, 29]). Considering the primitive pattern of budding in *Stephanella* as an early branch of Phylactolaemata [14], it is possible that it has retained the early or the original budding mode, and that other Phylactolaemata have reduced it.

**Fig. 2:**
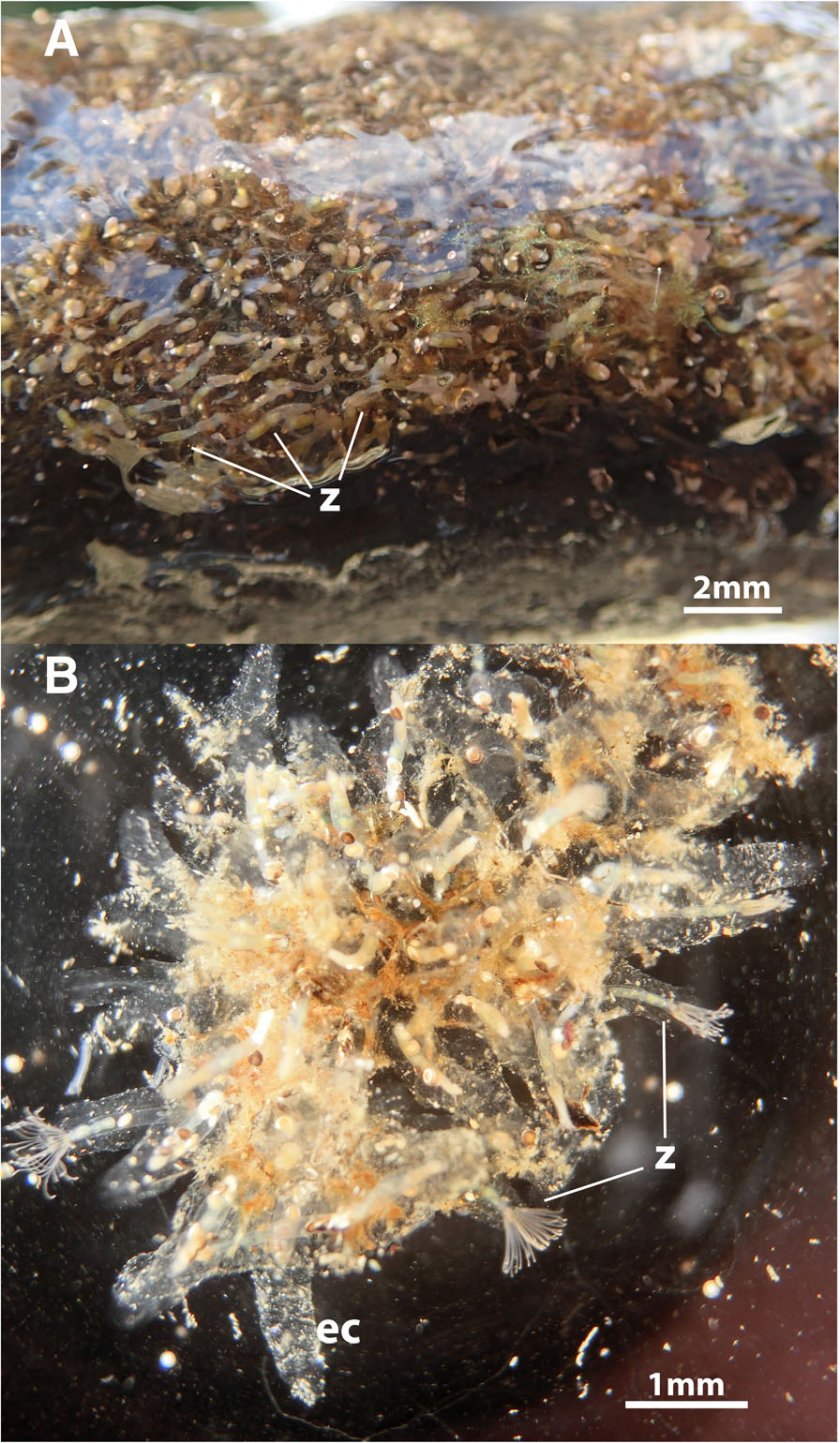
Comparison of the colony of *Stephanella hina* in and out of the water, showing the non-supportive and flexible ectocyst. **a** *Stephanella hina* colony on a piece of wood taken out of the water. Note the collapsed appearance of the colony with the zooids lying flat on the substrate. **b** Piece of a colony submerged into water. Note the upright position of the zooids extending vertically from the substrate. Abbreviations: ec – ectocyst, z – zooid

**Fig. 3:**
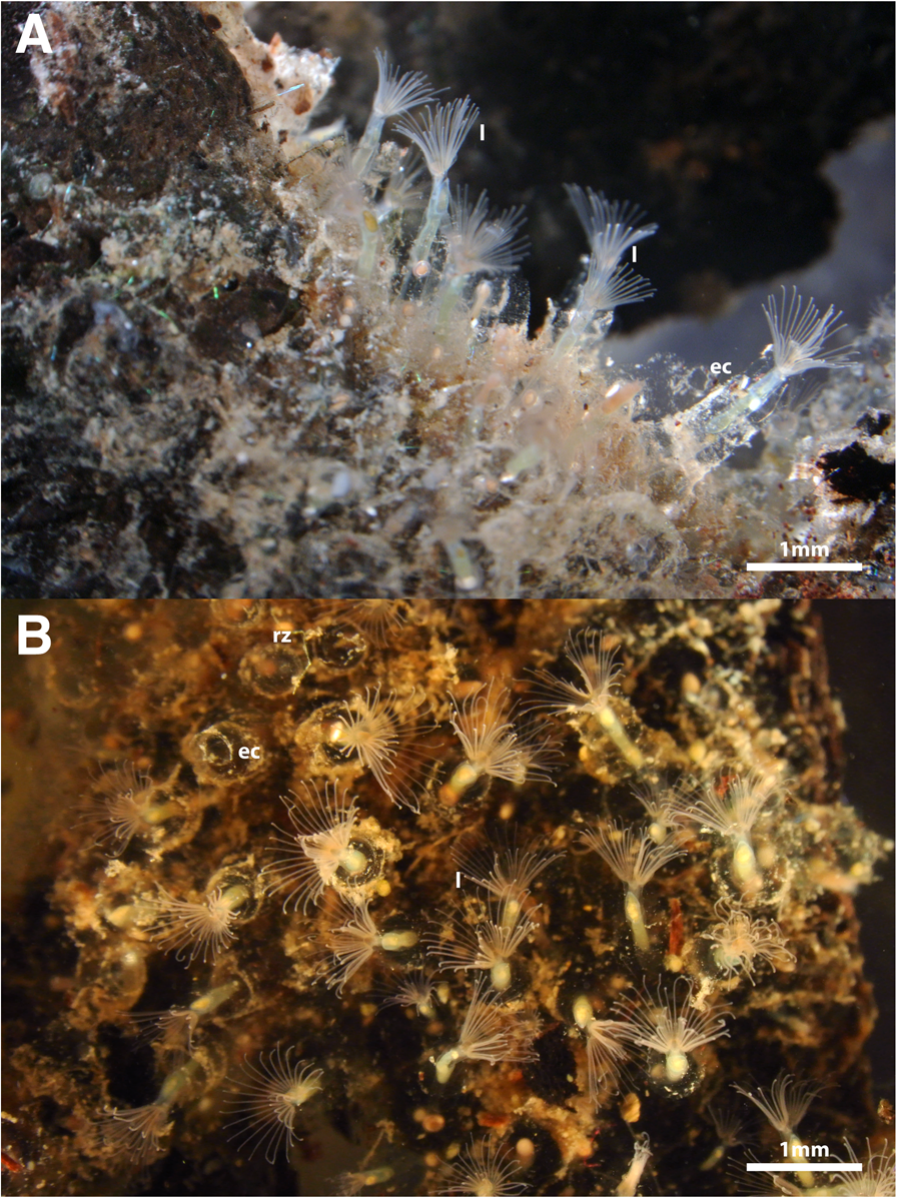
General morphology of colonies of *Stephanella hina*. **a** Lateral view of a colony, showing several individual zooids extending their lophophores into the water column. Note that the polypides extend vertically from the substrate. The transparent ectocyst can only be discerned by particles adhering to it. **b** Top view of a colony showing the arrangement of the individual zooids within the colony and the typical horseshoe-shaped lophophore. Abbreviations: ec- ectocyst, l – lophophore, rz – retracted zooid

**Fig. 4:**
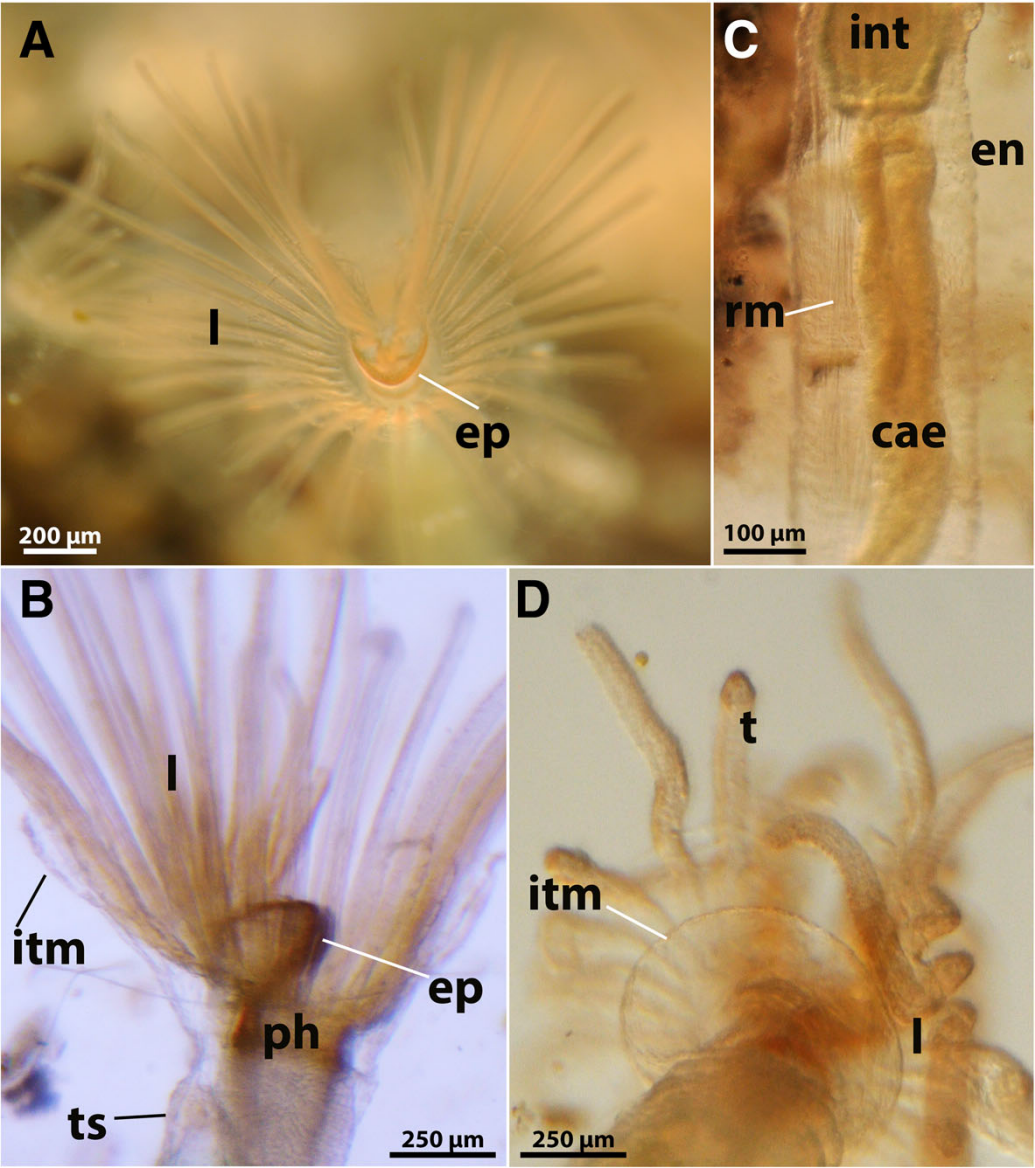
Details of a single zooid of *Stephanella hina*. **a** View of the lophophore with the horseshoe-shaped tentacle arrangement and the epistome protruding over the mouth opening. Note the orange tinge of the epistome. **b** Lateral view of the lophophoral base, showing the evident coloration of the epistome as well as the pharynx. Note the intertentacular membrane on the outer side of the lophophore. **c** Lateral view through the transparent ectocyst showing parts of the digestive tract as well as retractor muscle strands traversing the visceral coelom. **d** View from the basal side of the lophophore and the intertentacular membrane extending from the lophophoral base towards the outer area of the tentacles. Abbreviations: cae – caecum, en . endocyst, ep – epistome, int – intestine, itm – intertentacular membrane, l – lophophore, ph – pharynx, rm – retractor muscle, t – tentacle, ts – tentacle sheath

**Fig. 5:**
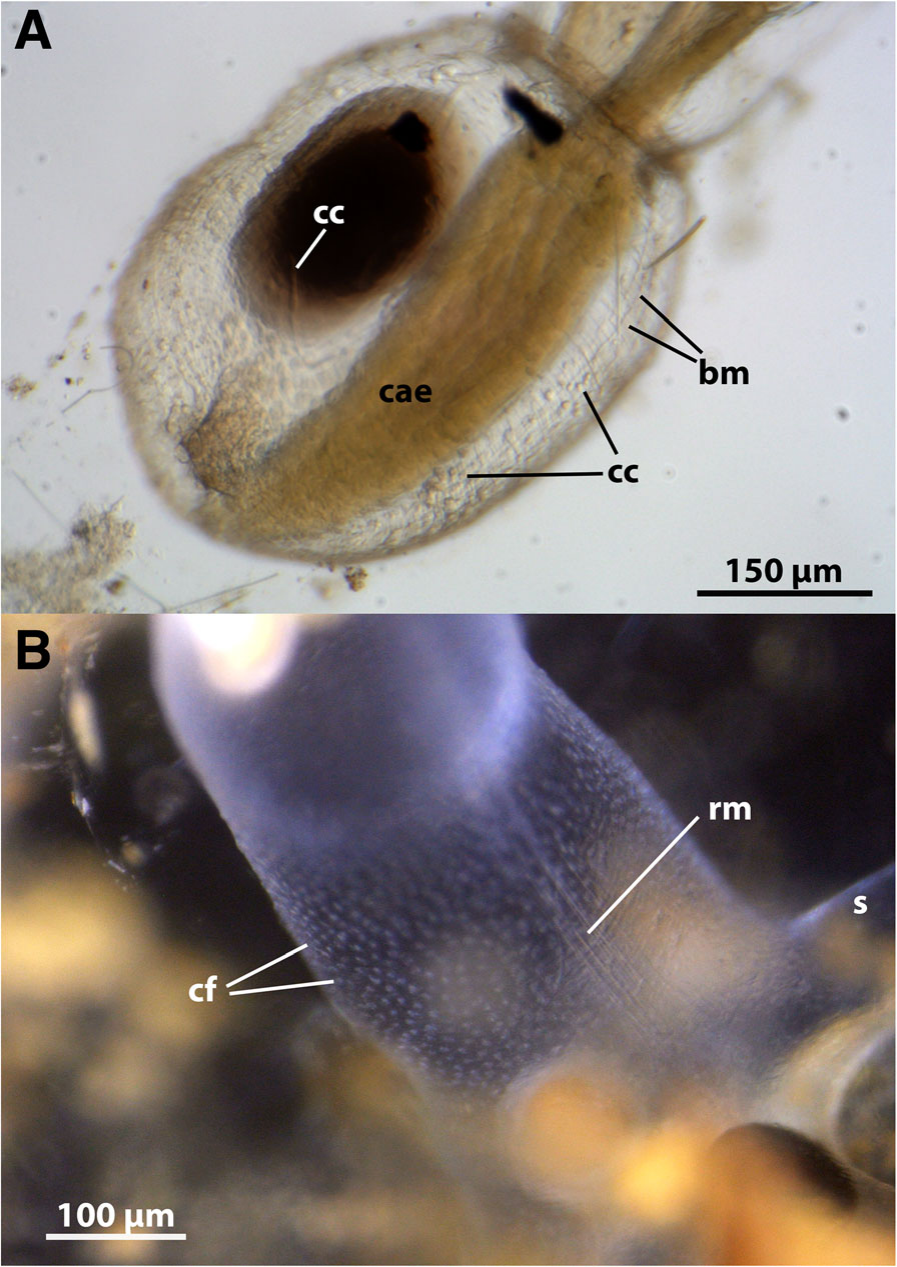
Coelomic structures of *Stephanella hina*. **a** Lateral view of a solitary zooid of *Stephanella hina*, showing body-wall musculature as well as coelomocytes within the coelomic cavity. **b** Lateral view of the proximal part of a zooid, showing the regular arrangement of ciliary fields of the peritoneal lining of the coelomic cavity as well as strands of retractor muscle fibres traversing the same. Abbreviations: bm – body-wall musculature, cae – caecum, cc – coelomocyte, cf – ciliary fields of ther peritoneal epithelium, rm – retractor muscle fibres, s - stolon

**Fig. 6:**
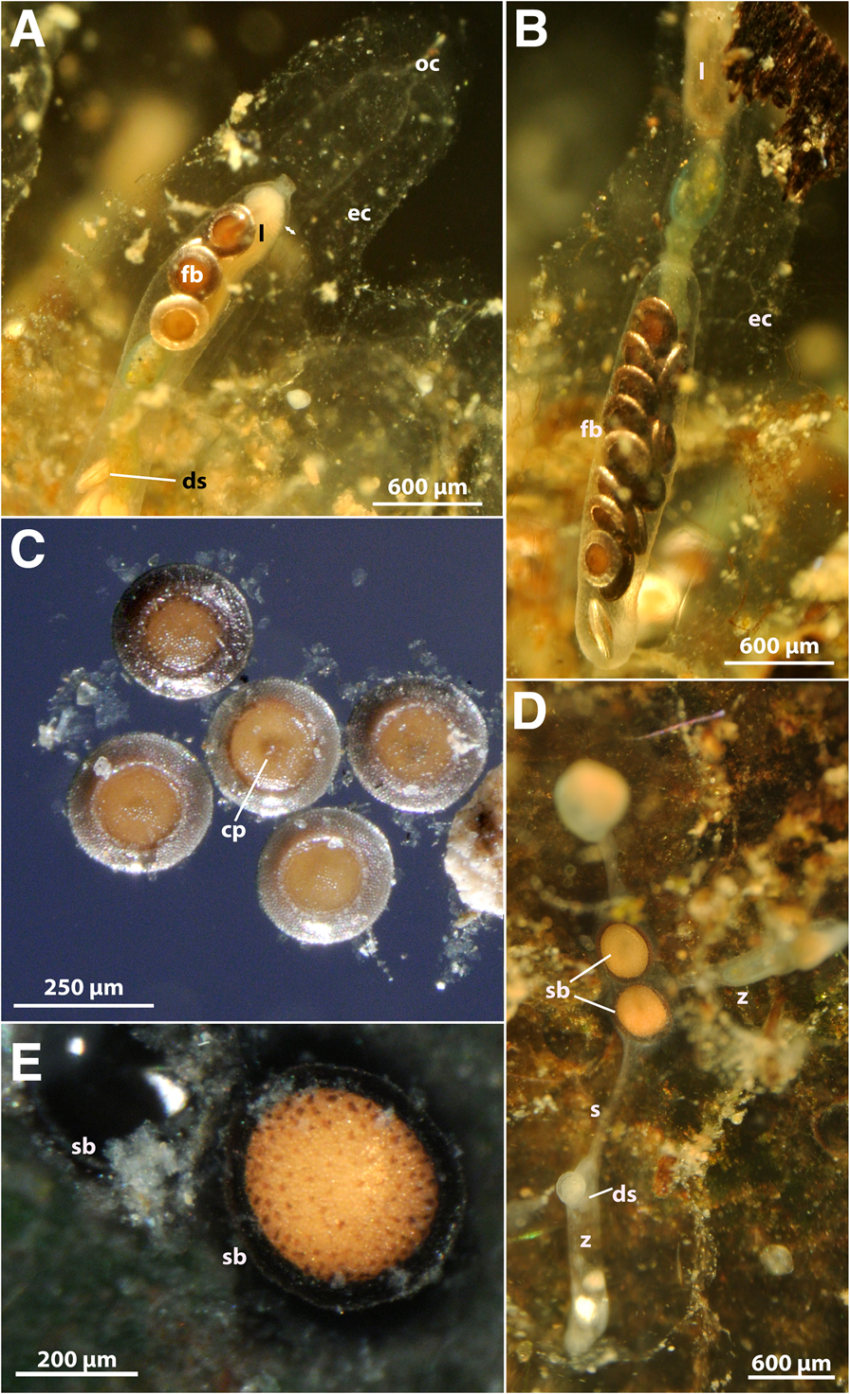
Statoblasts of *Stephanella hina*. **a** Statoblasts and developing statoblasts in a retracted zooid. Note the distance between the jelly-like cystid and the zooid (double arrow). **b** Single zooid with 14+ floatoblasts in the proximal cavity behind the zooid. **c** Detail of expulsed floatoblasts on the water surface. The floatoblasts are circular and show a darker annulus for floating and an inner orange fenestra, which contains the germination mass. Note a darker spot/central protuberance in the middle floatoblast indicating the deutoplasmatic side (ventral valve) of the statoblast. **d** Sessoblasts located in a distinct part of the colony not directly associated with a zooid but merely with stolons. The stolon-like processes interconnect individual zooids of the colony. **e** Detail of a sessoblast. The sessoblast is firmly attached to the substrate and lacks a floating annulus. Note on the left side sessoblast-remains. In the latter only the attaching cementus is present, indicating the probability that the frontal valve of the sessoblast was mechanically removed. Abbreviations: cp – central protuberance, ds – developing statoblast, ec – ectocyst, fb – floatoblast, l – lophophore, oc – opening of the ectocyst tube, s – stolon, sb – sessoblast, z –zooid


The main part of each zooid extends vertically from the substrate as a long slender tube that carries all the major components of the polypide, i.e., the digestive tract and the tentacle crown, the lophophore, which is used for filter-feeding (Figs. 2 and 3, see also Additional file 1: Video S1). From the lophophoral base, the intertentacular membrane extends towards the abfrontal side of the tentacles. It is rather short and surrounds the whole lophophore equally (Fig. 4b and d, see Additional file 2: Video S2). The tentacles, the epistome and the distal part of the pharynx show a reddish-brownish hue, which is more pronounced when animals are retracted and tissues are much more compact (Fig. 4a, b and d, see Additional file 2: Video S2, Additional file 3: Video S3, Additional file 4: Video S4). This coloration has been previously described by several authors [16, 18, 21]. The tentacle tinge is reportedly lost after two days of culture in pure tap water [16]. The digestive tract consists of the common parts found in other bryozoans, i.e. pharynx, oesophagus, cardia, caecum, intestine and anus. In our samples the digestive tract showed a blue-green coloration in most cases (Additional file 3: Video S3, Additional file 4: Video S4, Additional file 5: Video S5). The color of the digestive tract is commonly influenced by the food source, but the exact food contents have not been analyzed so far in *S. hina*. Previously, the coloration of the stomach was described as yellowish-green [21] to pale brown [18]. Retraction of the polypide is effected by a paired retractor muscle that emanates from the basal side and inserts at the lophophoral base and parts of the digestive tract (Figs. 1c and 4c, see Additional file 3: Video S3, Additional file 4: Video S4, Additional file 5: Video S5, Additional file 6: Video S6). The general transparency of the animals allows ready observation of the contraction and relaxation of these muscles. Contraction of the regular body wall musculature increases pressure within the coelomic cavity and along with relaxation of retractor and apertural muscles pushes the polypide out of the cystid. The body wall musculature can be observed in live specimens (Fig. 5a). The funicular cord runs from the proximal pole of the caecum to the body and carries the anlagen or fully differentiated free-floating statoblasts, the floatoblasts (Fig. 6). Floatoblasts in *S. hina* are circular in shape (Fig. 6a–c). At the basal side, sessoblasts are commonly attached to the substrate (Fig. 6d and e). These often occur in certain interzooidal widenings of the stolonal network, and rarely exceed 2–3 in number. The situation thus resembles that of other phylactolaemate bryozoans [13, 30]. So far, no gonads of either sex have been encountered. A single previous description found well-developed testes and some growing ovaries in very young colonies in December at 5 °C water temperature, but could not follow its subsequent development due to frozen ponds and lakes [20].

Additional file 1: Video S1. http://phaidra.univie.ac.at/o:440977. Video showing a colony of *Stephanella hina* with extended lophophores that are manipulated with a needle to show the retraction process. Note the soft transparent ectocyst that extends vertically from the substrate and gives only little support. (MOV 626 mb)
Additional file 2: Video S2. http://phaidra.univie.ac.at/o:440825. Top view of an extended zooid of *Stephanella hina*. Between the individual tentacles of the lophophore lies the intertentacular membrane surrounding the outer margin. The epistome can be seen by its reddish hue. At about 30 s into the video, the animal retracts into its jelly-like tube. Towards the end of the clip, the lophophore is everted again. (MOV 352 mb)
Additional file 3: Video S3. http://phaidra.univie.ac.at/o:440987. View into the proximal region of a zooid of *Stephanella hina*. The caecum has a greenish hue. To its right side the retractor muscle fibres are visible. From the proximal point of the caecum the funiculus runs in parallel direction to the retractor muscle fibres towards the body wall. At 1:45, mechanical stimulation leads to the retraction of the animal. The retracted lophophore, the epistome as well as anterior part of the pharynx have a reddish hue. Following retraction, the animal slowly expands again. The hindgut contains undigested food particles. Stretching of the retractor muscles can be clearly seen during the protrusion process. (MOV 794 mb)
Additional file 4: Video S4. http://phaidra.univie.ac.at/o:441003. The retraction process of *Stephanella hina*. A strong mechanical stimulus leads to the retraction of the polypide into the transparent ectocyst. The lophophore on the top side is readily distinguishable by its reddish hue, whereas most of the digestive tract shows shades of green and blue. Halfway into the video, the polypide has protruded again and unfolded its tentacle crown. A second retraction-protrusion cycle is seen in the second half of the video. (MOV 366 mb)
Additional file 5: Video S5. http://phaidra.univie.ac.at/o:440826. Feeding zooid of *Stephanella hina*. Particles are seen directed towards the mouth opening at the centre of the tentacle crown. Ingested particles are pumped up and down via muscular contractions within the caecum, seen on the far right-hand side. (MOV 399 mb)
Additional file 6: Video S6. http://phaidra.univie.ac.at/o:440821. Video of the proximal area of a solitary zooid of *Stephanella hina*. The body cavity is filled with numerous coelomocytes that circulate via ciliary beating within the body cavity. On the left side, the retractor muscles are apparent, stretching and shortening during the retraction process. (MOV 232 mb)


### The ‘cystid’ of *Stephanella hina*

The vertically extending tubes containing the polypides are embedded into a copious amount of the jelly-like, transparent ectocyst. Two different variations of this ectocyst were encountered: either it forms a connecting mass between the extended polypides, or each polypide is surrounded by its own tube, which is not connected to neighbouring ectocysts. Particularly in the latter condition, the cystid is not a firm structure that maintains the general structure of the colony, but appears to be more buoyant (Figs. 2b and 3, Additional file 1: Video S1). This is most evident when colonies and their substrate are removed from the water, which results in a collapse of the colony, resulting in a flattened appearance (Fig. 2a). The soft jelly-like and barely self-supportive form of this ectocyst was previously recognized [17, 18], whereas the first more dense form has not been reported. No distinct environmental cues are known for different cystid structure among colonies, especially since colonies were collected from the same localities and sometimes also at the same time of the year.

As described above, the ectocyst is for the most part transparent – in general, however, this ‘cystid’ has sticky properties and particles floating in the water column easily adhere to it as previously described by Oka [21] (Figs. 2b and 3a; Additional file 3: Video S3). Cystid structures in the non-calcified Phylactolaemata are either gelatinous as in most of the larger forms (Pectinatellidae, Cristatelllidae, Lophopodidae) or sand-encrusted to chitinous in the smaller, rather branching representatives (Plumatellidae, Fredericellidae) [13, 31]. The ectocyst in *S. hina* is different from that of the above-mentioned taxa, and is probably best characterized as jelly-like. It is most comparable in appearance and mechanical properties to the protein/glycoprotein jelly-coats of frog embryos [32].

**Fig. 7:**
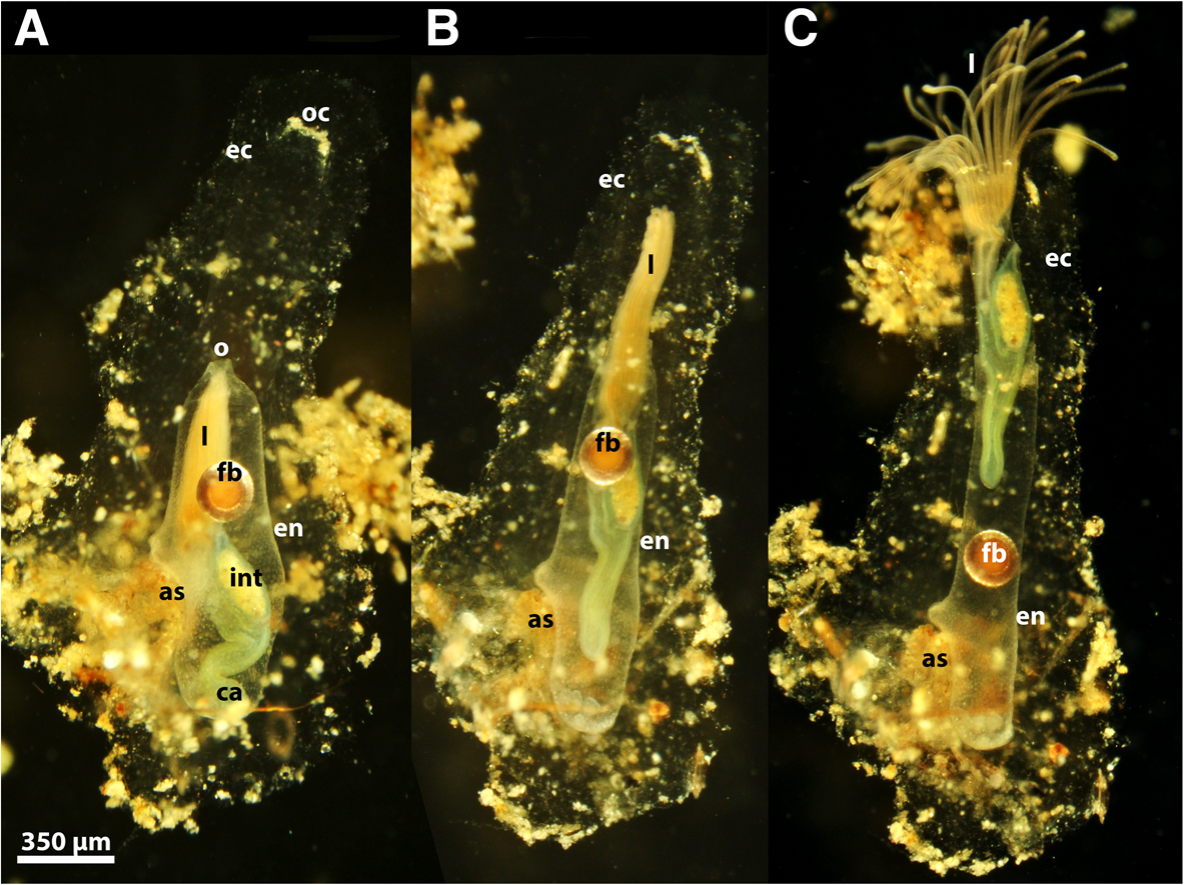
The retraction process of an individual zooid of *Stephanella hina* in regard to the ectocyst. **a** Completely retracted zooid. **b** Mid-retracted/extended zooid and **c** protruded zooid. Note that in all images there is a distinct gap between the endocyst and the ectocyst, and that the endocyst slides past the ectocyst during the retraction process. Abbreviations: as – cup-shaped attachment site, ca – caecum, ec – ectocyst, en – endocyst, fb – floatoblast, int – intestine, l – lophophore, o – orifice, oc – opening of the ectocyst tube


In all bryozoan zooids the cystid consists of an inner, living endocyst (the body-wall with the outer epidermis and inner peritoneum) and an extracellularly secreted cuticle, the ectocyst or sometimes called periostracum (see eg. [33] for phylactolaemates and [34] for calcareous taxa). These two components are commonly attached firmly to each other, especially in the calcified Stenolaemata and Cheilostomata. Analysis of the retraction process in *Stephanella hina* shows that the endocyst is for the most part not attached to the ectocyst and thus actually not a cuticle of the endocyst but a tube surrounding the zooid (Fig. 7). During the retraction process, the endocyst is quite distant from the ectocyst, leaving a considerable gap (Fig. 7, Additional file 7: Video S7). This kind of ectocyst is thus unique among Phylactolaemata and Bryozoa in general and superficially resembles tubes of other filter-feeding lophotrochozoans (e.g., tube-forming polychaete annelids and phoronids).

Additional file 7: Video S7. http://phaidra.univie.ac.at/o:440992. Mechanical tapping with a needle of a zooid of *Stephanella hina*. At the beginning only a short contraction into the distal part of the ectocyst is seen. The gap between the polypide and the ectocyst is clearly evident. In latter parts of the video only strong mechanical stimuli resulted in a pronounced retraction, in which the gap between the body-wall and the ectocyst becomes even clearer. (MOV 172 mb)


### Coelomocytes

Due to its transparency, internal structures can readily be observed. One very obvious feature in *Stephanella hina* is commonly elongated to roundish coelomocytes in the body cavity of the animals (Fig. 5a, see Additional file 6: Video S6, Additional file 8: Video S8 and Additional file 9: Video S9). Their amount and density seem to be highly variable and range from very few to several dozens. Cues affecting their abundance are unknown. Ciliary tufts regularly situated at the inner peritoneal layer (cf. [30, 33]) create a current in the coelomic fluid that circulates the coelomocytes within the coelomic cavity (Fig. 5b). Previously, up to nine different coelomocytes were categorized in the Phylactolaemata [35]. None have been described as elongated/rod-like cells as found in *S. hina* (see Additional file 6: Video S6, Additional file 8: Video S8 and Additional file 9: Video S9). Coelomocytes in bryozoans appear to play different important roles that remain poorly understood. They appear to originate as detaching cells from the peritoneal layer of the body-wall. Especially vital dye experiments indicate that some of these cells have excretory functions [35–37], whereas some were commonly termed ‘leucocytes’ or ‘lymphocytes’. An immunological function, however, could not be proven so far. Particular excretory organs/osmoregulatory organs such as proto- or metanephridial systems are absent among bryozoans (cf. [30]); their function seems to be taken over by coelomocytes, as has been described for marine bryozoans [38, 39].


Additional file 8: Video S8. http://phaidra.univie.ac.at/o:440823. View into the body cavity of *Stephanella hina* showing the yellowish caecum. Around the caecum, several coelomocytes are visible circulating within the body cavity. Note also the numerous dark and roundish anlagen of the floatoblasts. Towards the attachment site of the funiculus on the right-hand side, the anlagen are younger and less opaque than the more differentiated ones to the left. (MOV 215 mb)
Additional file 9: Video S9. http://phaidra.univie.ac.at/o:440817. Coelomocytes floating and circulating within the body cavity of a solitary zooid of *Stephanella hina*. (MOV 70.5 mb)


In the current investigation, a few cases of the expulsion of coelomocytes were observed from specimens containing numerous coelomocytes (Additional file 10: Video S10). This phenomenon was previously documented in a few other phylactolaemates [40–42] and occurs via the vestibular pore at the anal side of the vestibular wall at the orifice. The vestibular pore is a not well-recognized structure which in addition to the expulsion of coelomocytes is also the point where statoblasts as well as sperm are commonly liberated [13, 23, 40, 42–44]. Only recently the pore was morphologically confirmed in the phylactolaemate *Lophopus crystallinus*. In the latter, muscles are present at the pore that enable active closure after the release of substances [42]. It is highly likely that *S. hina* and other phylactolaemate species possess such a pore, but this needs to be confirmed morphologically on a broader scale. Gymnolaemate bryozoans possess a comparable pore, the supraneural pore, which seems, at least partially, to fulfill similar functions. However, it is located at the lophophoral base rather than in the vestibular wall [45].


Additional file 10: Video S10. http://phaidra.univie.ac.at/o:440820. View of a retracted zooid of *Stephanella hina*. The surrounding ectocyst is transparent with some superficial particles attached to it. Distally lies the orifice which leads to the vestibulum. Coelomocytes are present in the body cavity of the zooid. At the beginning of the video, coelomocytes are expelled, probably via a vestibular pore. (MOV 239 mb)


### Experimental observations

#### De-colonisation of colonies

Some of the sample material contained single zooids not connected with others; these were thus clearly solitary. Later observations showed that this resulted mostly from the disruption of the stolon during removal of the zooids from the substrate. Consequently, individual zooids were excised from the colony to observe whether this would affect the life of the individuals. We found that de-colonisation does not seem to have any negative effect. Separated zooids that contain fully differentiated floatoblasts generally float on the top of the water column.

Considering that separation of the individuals or a colony did not seem to affect the future life of the colonies raises questions about how well zooids are integrated into colonial communication. For most bryozoans, colonial integration of the individuals is assumed (i.e. the assumption is that individuals communicate in some way with other zooids of the colony, particularly in response to threats such as predators). For this purpose a colonial nervous system is present within the body-wall (see [46]). In the more advanced and integrated taxa Stenolaemata and Gymnolaemata, the colonial plexus is localized in more direct patterns than in the Phylactolaemata, in which the nervous system in the body-wall consists merely of a diffuse plexus [30, 46–49]. Since information on the nervous system in *Stephanella hina* is lacking, the extent of neuronal integration of zooids is not known, but it is clear that zooids communicate by stolon-like connections across longer distances than in other freshwater bryozoans. Numerous observations in which individual zooids were tapped to provoke retraction found no effect on neighboring zooids (see Additional file 11: Video S11). This is not uncommon, especially for freshwater bryozoans, where stimulation of individual zooids does not necessarily lead to the retraction of more than just the affected zooid. Instead, (mechanical) stimulation of the cystid wall results in simultaneous retraction [50]. Due to the lack of a firm connection of the soft ectocyst to the endocyst, cystid vibrations often do not result in a colonial retraction process. Also, in some cases removing individual zooids from the colony did not result in any defensive response in other zooids of the colony, whereas wounding has been found to be a common stimulus for retraction in other phylactolaemates [50]. Suffice to say, this appears to be a rather ineffective protective mechanism. Moreover, *S. hina* colonies commonly do not react to regular shifts in the water column; regular movement of the dishes containing the colonies did not cause the zooids to retract, but rather caused the vertically standing tube-like extensions of the zooids, including the jelly-like ectocyst, to follow the motion of the water. This gave the colony the appearance of ‘headbanging’ tentacle crowns (see Additional file 12: Video S12). Currently, reduced predation during the cold months appears to be the only explanation for why zooids appear rather unresponsive to such stimuli. As aforementioned, *S. hina* is one of the few species that occurs in winter. Perhaps lower temperatures also lead to a lower metabolism in these animals, and thus slower response times. Alternatively, or additionally, predation risk may be significantly decreased during colder times in freshwater.


Additional file 11: Video S11. http://phaidra.univie.ac.at/o:448565. Tapping of individual zooids of *Stephanella hina*. Note the jelly-like appearance of the ectocyst surrounding individual polypides. Note also that stimulation of single zooids does not affect neighboring zooids. (MOV 626 mb)
Additional file 12: Video S12. http://phaidra.univie.ac.at/o:440976. *Stephanella hina* colony exposed to rapid water flow changes by vigorously shaking the dish with specimens. Rapid movements shake the polypides without retraction, a movement similar in appearance to ‘headbanging’. (MOV 132 mb)


#### Ectocyst removal and cystid evolution

As described above, the ectocyst is for the most part not firmly attached to the body-wall; nonetheless, the retraction process functions properly. Investigation of de-colonized, solitary forms shows that parts of the basal wall form a cup-like structure that connects with the ectocyst. This is the area at which the retractor muscle fibres attach, and thus represents the support for the retraction process. This limited attachment of the body-wall to the ectocyst gave the opportunity to release zooids from their ectocyst hull (Additional file 13: Video S13). Severing the attachment site, in most cases with a pair of forceps, gave rise to zooids devoid of an ectocyst. The animals readily survived this treatment and continued feeding. Tapping animals that lack an ectocyst does not cause retraction of the tentacle crown, only a longitudinal contraction of the trunk as, in such individuals, the retractor muscle has no support from the area normally attached to the substrate (Additional file 14: Video S14). However, such animals are capable of restoring/regenerating their ectocyst coating. At first, the new ectocyst is produced on the basal site at the attachment of the retractor fibres in order to enable the typical retraction process. The ectocyst renewal proceeds distally towards the tentacle crown that protrudes into the water column. Mechanical stimulation of such specimens leads to partial contraction of the trunk (Additional file 15: Video S15).


Additional file 13: Video S13. http://phaidra.univie.ac.at/o:440979. Removal of the ectocyst from a solitary zooid of *Stephanella hina*. Note that during manipulation there is only a single attachment point towards the ectocyst, i.e. the area where the retractor also inserts. (MOV 140 mb)
Additional file 14: Video S14. http://phaidra.univie.ac.at/o:440824. The retraction process in a single zooid with the ectocyst removed of *Stephanella hina*. Due to the absence of a fixed point on the ectocyst, contraction of the retractor muscle leads to a shortening of the trunk. Note that, due to its transparency, the two retractor muscle bundles are quite evident. (MOV 224 mb)
Additional file 15: Video S15. http://phaidra.univie.ac.at/o:440828. Solitary zooid of *Stephanella hina* having the ectocyst tube removed on the previous day. The basal side on the left has formed attachment to a newly secreted ectocyst. Triggering the retraction response now leads to a shortening of the trunk and much more progressed retraction than without ectocyst. (MOV 304 mb)


Experimental ectocyst removal was previously conducted only in the lophopodid *L. crystallinus* [51], in which colonies also survived the procedure without great harm. The ectocyst in *L. crystallinus* in general is just a thin gelatinous layer that is in rather loose connection with the endocyst [40]. With the current position of stephanellids and lophopodids, this may imply that the original ectocyst was not a proper cuticle attached to the body-wall, but merely a tube-like, semi-adhering protective secretion. The lophopodid genus *Lophopodella* commonly has a thin layer of ectocyst (Hirose pers. obs.), whereas the condition among the other lophopodid genus *Asajirella* hast not been analyzed recently. In the latter it appears to be more massive and gelatinous [52, 53]. It is definitely present in copious amounts on the basal side (the side facing the substrate), but it is not clear to what extent it is present on the frontal side facing the water. *Pectinatella magnifica* (sole species of Pectinatellidae) also produces copious amounts of gelatinous ectocyst, but only the basal side is covered by ectocyst, whereas the frontal one is naked [52]. Likewise, *Cristatella mucedo* (sole representative of the Cristatellidae) also produces only a thin ectocyst at its creeping sole towards the basal side [51, 52]. Pectinatellidae and Cristatellidae are generally regarded as closely related and probably constitute sister-groups [14]. *Cristatella* has motile colonies through the whole life-stages, whereas *Pectinatella* colonies are sessile gelatinous masses and only young colonies are motile [54–56]. Consequently, the three phylactolaemate families Lophopodidae, Cristatellidae and Pectinatellidae are motile, at least at one point during their ontogeny [56]. A rigid and firm ectocyst is thus obstructive for any movement. It appears that only in the most speciose family, the Plumatellidae, the separation of the ectocyst is not possible; the same applies to the Fredericellidae. However, in the latter it should be noted that zooids are capable of detaching from the cystid and leave the colony in form of swimming zooids under unfavorable conditions [57]. Given that the latter two families are currently considered as the derived form [14], it is likely that the phylactolaemate ancestor was a semi-motile, gelatinous animal with a rather loose ectocyst. *Stephanella hina* appears to be somewhat intermediate between a motile and strictly sessile form, since it is definitely sessile, but almost shows no connection to the loose ectocyst.

### The *Stephanellidae*

The present study confirms that *Stephanella hina* is indeed a unique species among the Phylactolaemata. With its typical features, such as the colony structure, the jelly-like ectocyst and the mostly missing connection between endo- and ectocyst, it is clear that this species cannot be assigned to the Plumatellidae as it has been in previous studies (e.g. [17, 58]), but merits a separate family Stephanellidae as originally proposed by Lacourt [15]. Besides the clear distinguishing features presented above, Stephanellidae is, together with the Plumatellidae, the only phylactolaemate family to produce sessoblasts (sessile dormant stages/statoblasts). However, the formation of the sessoblast is essentially different in *S. hina* than in all plumatellids studied [18, 19]. In plumatellids the basal side of the sessoblast, attached to the substrate, derives from the so-called cystigenic side, whereas the opposite condition with the deutoplasmatic side forming the basal side is found in *S. hina* [18]. This substantiates separate placement into the Stephanellidae and indicates that sessoblasts probably evolved twice within the phylactolaemates. Finally, Fredericellidae and Plumatellidae show an oro-median gap within the intertentacular membrane [40], which all other families, including *Stephanella*, lack. The functional significance of this gap is not clearly understood. The lack of this particularly neglected feature again supports the placement of *Stephanella* into a separate family-level taxon. Accordingly, the features defining Stephanellidae are (i) stolon-like growth pattern with polypides mostly extending vertically from the substrate, (ii) lateral budding loci in addition to the normal buds produced on the oral side as in other Phylactolaemata, (iii) sessoblasts attaching to the substrate with the deutoplasmatic side, (iv) floatoblast circular and not oval as in Plumatellidae, (v) jelly-like ectocyst for the most part not in direct connection to the endocyst, (vi) lack of oro-median gap in the intertentacular membrane.

## Conclusions and outlook

*Stephanella hina* is an ideal candidate for experimental approaches, as the transparency of the ectocyst as well as the easy detachment of single individuals from the colony allows insight into internal processes, such as digestion and the enigmatic coelomocytes. Other phylactolaemate families, such as the Lophopodidae, Cristatellidae or Pectinatellidae, are also transparent, but their large size and compact colony shape impede proper microscopic investigation. Moreover, they are difficult to keep in the laboratory. We kept large *Stephanella* colonies in a simple, aerated bucket at room temperature in the lab without any special care for over a week. The colonies were still in very good condition, which indicates that laboratory culture should be possible without too much effort. Some of the smaller branching forms, such as plumatellids or fredericellids, are opaque in their native habitat, but in case of *Fredericella* can be rendered transparent under proper laboratory culture [59]. The latter is particularly interesting for use in parasitological investigations, since phylactolaemate bryozoans are definitive hosts of myxozoan parasites, which can be observed within the body cavity [60]. So far no parasitic stages are known for *Stephanella*, a fact that perhaps could be correlated to their life in the cold times of the year, but it might be an interesting aspect to study in the future. Some undulating worm-like objects were encountered in some zooids. Some of these appear to be slowly circulating elongated coelomocytes, whereas others might represent ingested particles that were not properly digested and traverse the digestive tract unharmed.

What potential does this species have for phylogenetic inferences of the Bryozoa? Taking this species as one of the earliest branches, if not the earliest branch among Phylactolaemata, the conditions found in *S. hina* are perhaps plesiomorphic. Bryozoa were often regarded being derived from worm-shaped organisms, perhaps similar to the Phoronida (e.g. [61]). Many filter-feeding annelids and also phoronids are capable of secreting protective tubes from the body-wall which the animals can retract into ([62–64] for Annelida) ([65] for Phoronida). The ectocyst of *S. hina* is more similar to these tubes rather than the firmly attached ectocyst (thus a proper cuticle) of other bryozoans. Filter-feeding worms that live in tubes commonly use the longitudinal musculature to retract into the tubes. Accordingly, where has the retractor muscle of bryozoans evolved from? The last attempt of an evolutionary interpretation regarded it as condensation and separation of longitudinal body-wall muscles (cf. [66]). This view was, however, biased by the ‘lophophorate’ concept, and took the phoronids as closest to the Bryozoa for granted. In this line of thought, the derivation of the retractor muscle appears likely from the prominent feather-like longitudinal muscle bundles found in phoronids. However, even without this bias, the derivation of the retractor muscle from a longitudinal body-wall musculature appears likely when a worm-shaped ancestor is assumed. The condition of zooids without an ectocyst in *Stephanella hina* also showed trunk contractions, similar to the shortening of a worm-shaped body due to contraction of a longitudinal body-wall musculature.

In the early branching lophopodids an epistome is lacking [33], which is traditionally considered to be one the phylactolaemate-specific characters (e.g. [10, 30]). Such a small flap-like protuberance over the mouth opening is also present in other ‘lophophorate’ phyla and was considered homologous among the three ‘lophophorates’. Future studies should deal with the structure of different organ systems such as the epistome in order to assess whether this character has indeed been present at the base of all Phylactolaemata or has evolved within this group. In addition, future field work should aim at finding sexually mature colonies as well as larvae, since any kind of sexual reproduction is currently unknown in *Stephanella*.
